# ROMA: Representation and Quantification of Module Activity from Target Expression Data

**DOI:** 10.3389/fgene.2016.00018

**Published:** 2016-02-19

**Authors:** Loredana Martignetti, Laurence Calzone, Eric Bonnet, Emmanuel Barillot, Andrei Zinovyev

**Affiliations:** ^1^Computational and Systems Biology of Cancer, Institut CurieParis, France; ^2^PSL Research UniversityParis, France; ^3^Institut National de la Santé et de la Recherche Médicale U900Paris, France; ^4^Mines ParisTechParis, France

**Keywords:** module activity, gene set, overdispersed pathway, coordinated pathway, gene expression, proteomics, transcription factors

## Abstract

In many analyses of high-throughput data in systems biology, there is a need to quantify the activity of a set of genes in individual samples. A typical example is the case where it is necessary to estimate the activity of a transcription factor (which is often not directly measurable) from the expression of its target genes. We present here ROMA (Representation and quantification Of Module Activities) Java software, designed for fast and robust computation of the activity of gene sets (or modules) with coordinated expression. ROMA activity quantification is based on the simplest uni-factor linear model of gene regulation that approximates the expression data of a gene set by its first principal component. The proposed algorithm implements novel functionalities: it provides several method modifications for principal components computation, including weighted, robust and centered methods; it distinguishes overdispersed modules (based on the variance explained by the first principal component) and coordinated modules (based on the significance of the spectral gap); finally, it computes statistical significance of the estimated module overdispersion or coordination. ROMA can be applied in many contexts, from estimating differential activities of transcriptional factors to finding overdispersed pathways in single-cell transcriptomics data. We describe here the principles of ROMA providing several practical examples of its use. ROMA source code is available at https://github.com/sysbio-curie/Roma.

## 1. Introduction

The current availability of high-throughput genomics techniques such as transcriptomics makes it possible to accurately measure molecular profiles of a biological system at multiple levels (Hawkins et al., 2010). Given the large amounts of quantitative data produced by these system-wide experiments, the interpretation of results in terms of cellular processes and pathways becomes a crucial issue. Dedicated integrative analyses are needed to synthesize and transform data into valuable biological insight (Hawkins et al., 2010).

Many biological and clinical applications require the comparison of samples from different conditions. The objective of the analysis often requires highlighting signaling pathways and transcriptional programs that distinguish between the compared conditions. A widely used approach in cancer genomics consists in comparing measurements at the single gene or protein level to identify potential indicators of a particular disease state (biomarkers) or driver genes causally linked to the tumor initiation and progression (Barillot et al., 2012). In recent years, it has become clear that in cancer and other systemic diseases the same pathways can be affected by defects in different individual genes and that molecular profiles of tumor samples are more similar at the pathway level than at the gene level (Wang et al., 2010). Application of pathway-based approaches in the analysis of genomic data can help capturing biological information that is otherwise undetectable by focusing on individual genes. The idea of pathway quantification is widely exploited to extract biological information from high-throughput data (Levine et al., 2006; Ramos-Rodriguez et al., 2012; Borisov et al., 2014).

**Figure 1:**
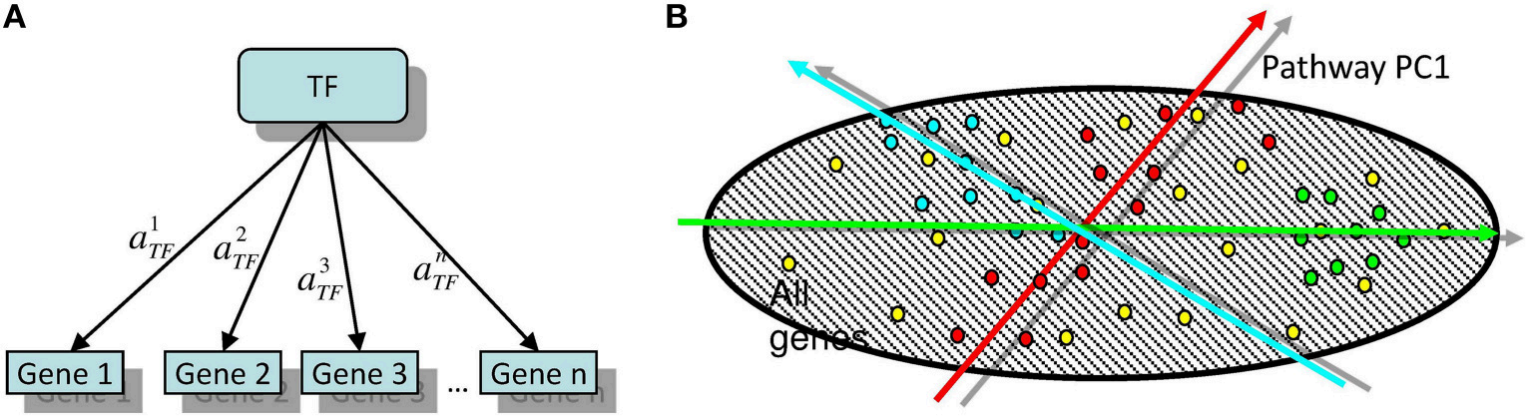
**(A)** The simplest linear model of gene regulation: expression of a gene is proportional to the activity of a transcription factor TF. **(B)** Illustration of two possible configurations of target genes in the global gene expression space. Here, the points of different color signify the genes participating in different modules. Red points are symmetrically overdispersed in both directions from the global data distribution center, while the green points are displaced with respect to the global data distribution center. Computing the principal component passing through the center of the global (represented by dashed area) data distribution allows quantifying both types of pattern in a similar and comparable way. Moreover, the position of the center of the global data distribution defines the reference point with respect to which the sign of the projection of a gene onto the principal component can be defined.

Here we propose an algorithm, released as a software, Representation Of Module Activity (ROMA), that was designed to address the issue of quantifying the activity of gene sets (further referred to as modules) characterized by coordinated gene expression. These modules can correspond to genes sharing the same functional annotations or regulatory motifs, genes belonging to the same pathway or genes forming a group of frequently coexpressed genes. The idea behind ROMA consists in quantifying module activity by computing the largest amount of one-dimensional variance across samples explained by the genes in the module (property of the first principal component or PC1). This is interpreted as a result of the action of a hidden factor on the expression of target module genes and variability in the activity of this factor in the studied collection of samples. This setting corresponds to the simplest linear model of gene expression regulation (for example, see Schreiber and Baumann, 2007; Figure 1).

ROMA implements several novel functionalities compared to existing related approaches. It allows determining genes within a group of genes contributing the most to the PC1 definition; it provides several alternative methods for PC1 computation, including weighted, robust and centered versions of principal component analysis; it estimates the statistical significance of the amount of variance explained by PC1 in two different ways; it distinguishes *overdispersed* and *coordinated* modules.

Here overdispersion of a gene set signifies that the amount of variance explained by PC1 computed for a dataset restricted to the genes from the set is significantly larger than for a random gene set of the same size. Coordinated gene set means that the spectral gap between the first and the second eigenvalues of the co-variance matrix computed for the restricted dataset is significantly larger than for a random gene set of the same size. Overdispersion signifies higher variability of a gene set even without increased correlations between genes. Coordination signifies relatively high degree of expression level correlation between genes in a gene set. Overdispersed set might be not coordinated: this is interpreted as simultaneous strong influence of several factors on the expression of the genes in the set. Coordinated set might be not overdispersed: this corresponds to a relatively weak but detectable activity of one single transcription or other factor on gene set expression. The most interesting and interpretable case is the case of simultaneous overdispersion and coordination of a gene set.

Naive quantification of the module activity frequently consists in computing the average or the median expression of the genes in the module in a given sample or, in opposite, relies on a single gene marker of module activity. ROMA is particularly suitable to model cases in which the different genes do not contribute similarly to the activity of the module, like the case in which some genes may be more important than others to define the activity of the module, or the case in which some genes are expected to negatively correlate with the activity of the module (e.g., p21, an inhibitor of the cyclin-dependent kinase complexes, may belong to a module of genes involved in the G1/S transition).

Several pathway quantification methods have been already proposed to recapitulate the activity of a module by computing the first metagene in the singular value decomposition (SVD) of the expression matrix restricted to the genes of the module (Tomfohr et al., 2005). In Bild et al. (2006) similar strategy was exploited in order to define the activity of several cancer-related pathways [MYC, RASA1 (RAS), SRC, Wnt/β-catenin and loss of RB function] on a large collection of human cancer transcriptomes. In Fan et al. (2016) the authors suggested the notion of “overdispersed pathway" in single-cell transcriptomic analysis framework such that the measure of activity in a set of genes is quantified by the statistical significance of the overdispersion explained by the first (weighted) principal component (PC1), computed for a set of single-cell transcriptomic profiles. Other methods have been developed for estimating module activity scores in individual samples such as single-sample extension of GSEA (ssGSEA) (Barbie et al., 2009) or OncoFinder (Borisov et al., 2014).

We illustrate the use of ROMA with four examples. In the first example, we quantify activities of several transcription factors (TFs) in metastatic and non-metastatic human colon tumor samples. In the second example, ROMA explores the transcriptional activity of modules in a comprehensive map of molecular interactions involving RB/E2F pathway in bladder cancer. The third application exploits ROMA to quantify transcriptional activity of targets for the oncogenic chimeric transcription factor EWS–FLI1 responsible of Ewing sarcoma initiation. Finally, we show an application of ROMA in the context of single-cell transcriptomic temporal profiling of myoblast differentiation (Trapnell et al., 2014).

## 2. Materials and methods

### 2.1. First principal component as the simplest uni-factor linear model of gene expression regulation

Let us consider the simplest model of gene regulation in which it is assumed that the expression of a gene *g* in sample *s* is proportional to the activity of one factor *F* (which can be a transcription or any other endogenous or exogenous factor affecting gene expression) in sample *s* with positive or negative (response) coefficient (Figure 1A):

(a) $$\begin{array}{l} Expression\left(geneg,samples\right)\approx \alpha_{g}^{F}Activity_{s}^{F}+B_{s}, \end{array}$$

where $\alpha_{g}^{F}$ is the coefficient of response of a gene *g* to the factor *F*, $Activity_{s}^{F}$ is the activity of the factor *F* in sample *s*, and *B*_*s*_ represents any sample-specific bias in measuring gene expression, affecting expression of all genes in sample *s* (*B*_*s*_ is analogous of the regression intercept in this linear model). In all further computations, we will assume that $\underset{s}{\sum }Expression\;\left(g,s\right)=0$ for all genes. Without this normalization, there is a possibility that the computed PC1 will only explain the variations in the basal gene expression (which is frequently the case). By applying double-centering of the gene expression matrix, containing genes in a gene set *G*_*i*_, i.e., making both $\underset{s}{\sum }Expression\;\left(g,s\right)=0$ and $\underset{g\in G_{i}}{\sum }Expression\;\left(g,s\right)=0$, one can achieve also *B*_*s*_ = 0. We do not suppose this normalization in the rest of this manuscript, because different gene sets can have different shift with respect to the center of the global distribution, hence, *B*_*s*_ = 0 can not be achieved for all gene sets at the same time.

Typically neither $Activity_{s}^{F}$ (activities of the factor in individual samples) nor $\alpha_{g}^{F}$ (the strength with which the factor *F* affects individual genes) are directly measurable. However, the simplest model fitting problem

(b) $$\begin{array}{l} \sum_{s}\text{​}\sum_{g}\text{​}{\left(Expression(gene\;g,samples)-\alpha_{g}^{F}Activity_{s}^{F}-B_{s}\right)}^{2}\text{​}\to \text{​}\min, \end{array}$$

with constraints

(c) $$\begin{array}{l} \sum_{g}{\left(\alpha_{g}\right)}^{2}=1,\sum_{g}\alpha_{g}=0 \end{array}$$

is solved by finding the PC1 of the expression dataset *Expression* (*g, s*), *g*∈*G*_*i*_, *s*∈*S* restricted to the genes from a selected gene set *G*_*i*_ over all sample set *S*. If the data set does not contain missing values, then $B_{s}=\frac{1}{\left|G_{i}\right|}\underset{g}{\sum }Expression\;\left(g,s\right)$. To find both $Activity_{s}^{F}$ and $\alpha_{g}^{F}$, one can apply the standard iterative SVD (Singular Value Decomposition) algorithm (e.g., see Gorban and Zinovyev, 2009), by starting with a random vector $Activity_{s}^{F}$ and computing $\alpha_{g}^{F}=\frac{\underset{s}{\sum }\;\left(Expression\;\left(g,s\right)-B_{s}\right)Activity_{s}^{F}}{\underset{s}{\sum }\;{\left(Activity_{s}^{F}\right)}^{2}}$. Then, the computed $\alpha_{g}^{F}$ are normalized to satisfy (c), and the new vector of factor activities is computed: $Activity_{s}^{F}=\underset{g}{\sum }\alpha_{g}^{F}Expression\;\left(g,s\right)$. The iterations are repeated until convergence. The constraints (c) are needed to guarantee convergence of this simple algorithm avoiding possible stretching or systematic drift of the $\alpha_{g}^{F}$ values.

Throughout the article, we will refer to a gene set *G*_*i*_ as “module” (accompanied by proper gene weights and signs if possible, as described below), where the biological interpretation of a “module" can be any functionally related list of genes, such as a set of direct targets of a transcription factor or other regulatory molecule, genes participating in the same signaling pathway as it is described in pathway databases, set of genomically co-localized genes, a set of genes containing the same motif for a transcription binding site, a set of co-expressed genes as a response to a particular perturbation, etc.

### 2.2. Principal component computation with weights or fixed center

Computation of the PC1 can take into account the a priori estimated relative importance of a gene *g* in the module *G*_*i*_. In order to achieve this, ROMA takes as an input the module descriptions which consist of a list of genes with a signed weight $w_{g}^{\left(G_{i}\right)}$ specified when possible (positive for “activators” and negative for “inhibitors” and undefined sign if the role of the gene is not known). The weights can be assigned only for some of the module genes with others being assigned the default 1.0 weight and undefined sign.

The computation of the principal components in ROMA is performed by the standard weighted SVD iterative algorithm as described in Gorban and Zinovyev (2009), where the weights for SVD are taken as the absolute values of the weights $\left|w_{g}^{\left(G_{i}\right)}\right|$ of the genes in the module. Introducing weights corresponds to generalizing the model fitting problem (d) to

(d) $$\begin{array}{l} \sum_{s}\sum_{g}|w_{g}^{\left(G_{i}\right)}|(Expression\;(geneg,samples)\\ \;-\;\alpha_{g}^{F}\;Activity_{s}^{F}-B_{s}{)}^{2}\to \min. \end{array}$$

Furthermore, in many cases, the activity of a module does not correspond to overdispersion of the module in the global gene expression space but to a shift of the genes in a particular direction (see Figure 1B). It is possible to quantify simultaneously this configuration of points and the overdispersed pattern using a simple modification of principal component computation such that the principal component would always pass through the center of the global distribution of points. This corresponds to the following modification of the initial linear model of gene regulation:

(e) $$\begin{array}{l} \sum_{s}\sum_{g}|w_{g}^{\left(G_{i}\right)}|{\left(Expression\;(g,s)\;-\;\alpha_{g}^{F}Activity_{s}^{F}\;-\;C_{s}^{fixed}\right)}^{2}\to \min, \end{array}$$

where $C_{s}^{fixed}$ is the global central point of the data distribution. In this case, we do not assume (c) and it might be that all α_*g*_s will possess the same sign (e.g., all targets being activated by a transcription factor).

We call this way of computing principal components as “PCA with fixed center.” It is used by default in ROMA, though standard PCA (d) can be also used.

### 2.3. Orienting principal components

In the standard principal component analysis, all components are computed with undefined orientation sign: there is an inherent mirror symmetry in the optimization problem (d) because the optimized function is symmetric with respect to $\alpha_{g}\to -\alpha_{g},Activity_{s}^{F}\to -Activity_{s}^{F}$ transformation. In ROMA we use the *a priori* information about the signs of genes in the module *G*_*i*_ to prefer one of two possible orientations of the PC1. We choose the orientation of PC1 for which

(f) $$\begin{array}{l} \underset{g\in W^{\left(G_{i}\right)}}{\sum }w_{g}^{\left(G_{i}\right)}\alpha_{g}^{\left(G_{i}\right)}>0, \end{array}$$

where $W^{\left(G_{i}\right)}$ is the set of genes in *G*_*i*_ for which both sign and weight are defined in the module description.

### 2.4. Computing robust first principal component

The computation of the PC1 can be affected even by a single outlier in the data set. In order to increase robustness of the PC1 computation, we apply here the “leave-one-out" cross-validation approach (Hastie et al., 2001). We compute the distribution of $L_{1}^{i}$ values where $L_{1}^{i}$ is the variance explained by the PC1 with the point *i* removed. The distribution $L_{1}^{i}$ is converted into a set of *z*-values, and all points with the absolute *z*-value bigger than *z*_*max*_ are removed from the dataset, where *z*_*max*_ is specified as a parameter (3.0 by default).

### 2.5. Estimating statistical significance of the variance explained for a module

The PC1 can be computed for any random set of genes, and it will assign the hidden factor activity in the samples for any randomly chosen gene set. In order to avoid overfitting, we perform an empirical statistical test estimating the probability of a module to be *overdispersed* (i.e., to explain in the PC1 more variance than expected for a random set of genes) or *coordinated* (i.e., to explain in the PC1 more variance compared to the second principal component than expected for a random set of genes). Let us denote by *L*_1_ the amount of variance explained by the PC1 and by *L*_2_ the amount of variance explained by the second principal component. It is important to notice that the randomly expected values of both *L*_1_ and *L*_2_ strongly depend on the size of the module for which it is computed. Therefore, we compute the empirical null distributions for values *L*_1_ and $\frac{L_{1}}{L_{2}}$ for *K* randomly chosen modules of the same size as the tested gene set.

In practice, there is frequently a need to test many module definitions. Estimating the null distribution for each tested gene set might lead to very expensive computations in terms of time. In ROMA, we do not compute the overdispersion significance scores for all possible module sizes, but instead we approximate them on predefined grid of size values. In order to rapidly estimate the significance of both overdispersion score (*L*_1_) and the coordinatedness score ($\frac{L_{1}}{L_{2}}$), we construct the null distributions for a selected representative list of module sizes. The representative module sizes are chosen to be uniformly distributed in the log scale between the minimal size of the module in the collection and the maximal module size. For computing the empirical *p*-value, the null distribution which is the closest one in terms of size in the log scale is chosen.

### 2.6. Data preprocessing for ROMA

The input format for gene or protein expression for ROMA is a tab-delimited text file with columns corresponding to biological samples and rows corresponding to genes or proteins. The first line is assumed to contain the sample identifiers while the first column is assumed to contain the non-redundant names of genes or proteins. In addition, ROMA can use description of samples also in tab-delimited text file format, in which the first row is assumed to contain the names of the features with which the samples are annotated and the first column will contain the names of the samples, in the same format as they are defined in the first row of the expression data table.

Optionally the input expression data can be centered or double-centered. If the data table contains missing values, they can be imputed using the approximation of the data matrix with missing values by a complete lower-rank matrix. For this, the user has to specify the rank *k*_*rank*_ of the approximative complete matrix. After this, *k*_*rank*_ principal components are calculated using the PCA algorithm able to work with missing data values (Gorban and Zinovyev, 2009). This PCA decomposition is used to construct the lower rank complete approximative matrix, from which the missing values in the initial data are imputed. For further computations, the completed initial data matrix of full rank is used.

### 2.7. ROMA implementation and workflow description

ROMA is implemented as a Java library which can be launched in command line. For computation of weighted PCA, and PCA with fixed center, ROMA exploits *vdaoengine* library. ROMA source code with instructions to build and run the application are available at http://github.com/sysbio-curie/Roma.

**Figure 2:**
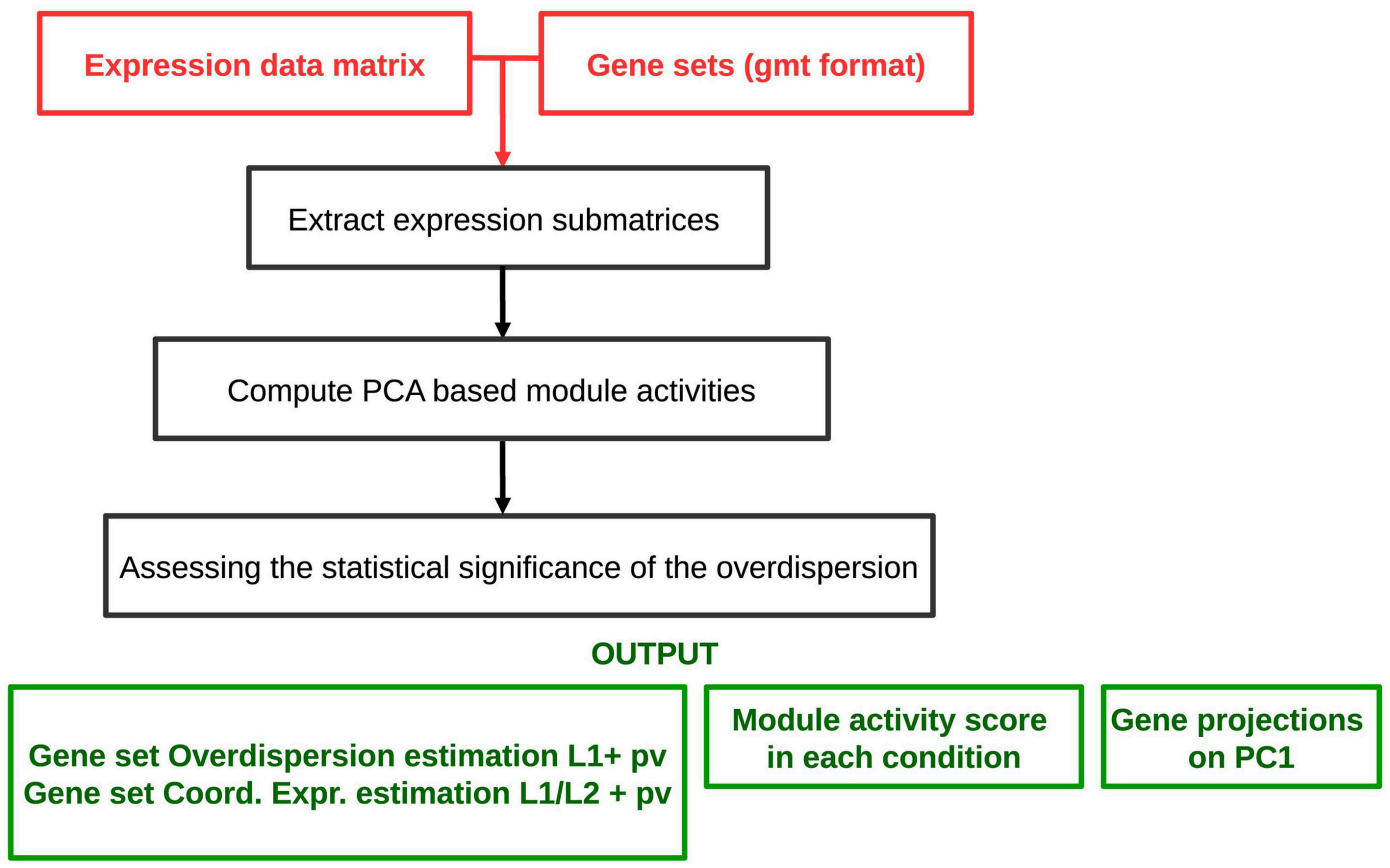
**Schematized workflow of the ROMA algorithm**.

The analysis workflow is schematized in Figure 2. The algorithm requires as an input a genome-wide expression data matrix and a gmt file with predefined modules. The analysis comprises a multistep procedure for (i) extracting expression submatrices corresponding to each module, (ii) quantifying robust PC1 based module activities and (iii) assessing the statistical significance of the *L*_1_ and $\frac{L_{1}}{L_{2}}$ values. ROMA provides as outputs different text files and tables including: a module score table with the overdispersion scores (*L*_1_) and the coordinatedness scores ($\frac{L_{1}}{L_{2}}$) with corresponding *p*-values for each module, a matrix file with rows containing the activity scores of each module across samples, a table for each module reporting the projections of genes in the PC1-PC2 space computed for a given module.

## 3. Results

As previously mentioned, typical scenarios for applying ROMA is to measure the activity of a transcription factor. It can also be applied in other cases, such as finding the activity of a kinase from phosphoproteomic data, or finding an abstract aggregated “activity" of a set of functionally related genes (such as genes belonging to the same pathway), assuming that overdispersed or coordinated behavior of the genes in the pathway is an indicator of its active state. We describe the application of ROMA to multiple case studies. In three of them, the biological information about the activity of the modules under study was a priori available and confirmed by ROMA results. The last case study shows an exploratory analysis by ROMA applied to single-cell RNA-seq data.

### 3.1. Notch, Wnt, and p53 pathways activity in human colon cancer

As a first case study, we applied ROMA to quantify the activity of Notch, Wnt and p53 pathways in invasive and non-invasive human colon tumors. In a previous study on a mouse model, p53 loss of function and Notch gain of function have been predicted to have synergistic effect in the induction of the epithelial to mesenchymal (EMT)-like phenotype (Chanrion et al., 2014). To investigate in human data the involvement of Wnt, p53, and Notch pathways in EMT induction, we used a publicly available gene expression dataset of human colon cancer samples from The Cancer Genome Atlas (TCGA) project (Muzny et al., 2012) and compared the activity scores of Notch, Wnt and p53 pathways in metastatic and non-metastatic samples. Genome-scale expression profiles of 121 tumor samples were used in our analysis.

**Figure 3:**
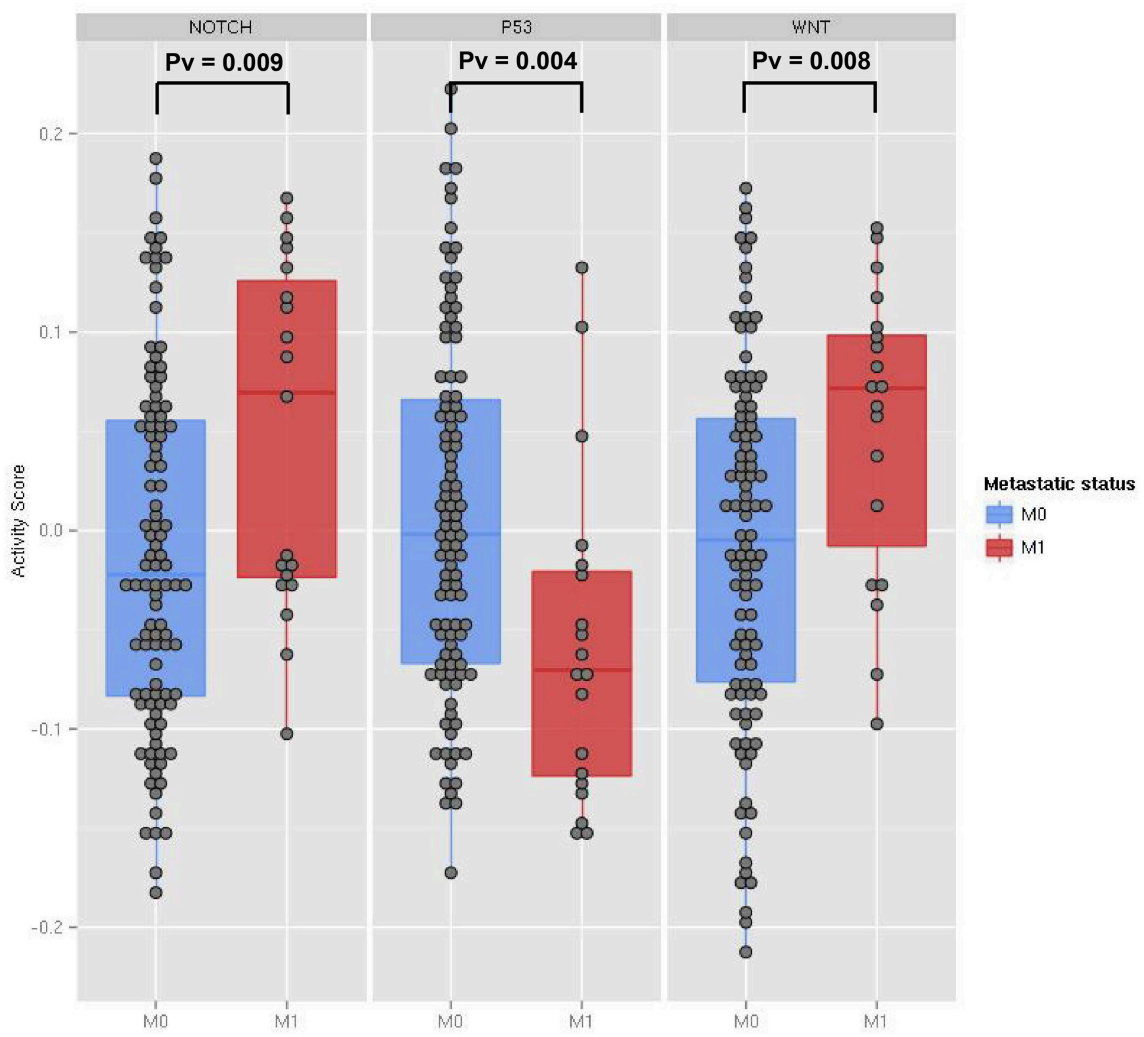
**The activity scores computed for the Notch, p53 and Wnt pathways in human transcriptome data from TCGA colon cancer samples**. The data points represent primary tumor samples grouped as non-metastatic (blue) and metastatic (red) according to the observation of distant metastases. *P*-values are calculated using the two-sample Kolmogorov-Smirnov test between the two groups.

Differential expression analysis of single genes involved in Wnt and Notch signaling pathways did not show significant changes between metastatic and non-metastatic tumors (see File S1). Thus, we investigated the involvement of these pathways by computing with ROMA the activity scores of their downstream target sets. Levels of pathway activity across tumor samples revealed that Notch and Wnt pathways were significantly activated, whereas the p53 pathway was downregulated in the metastatic compared to non-metastatic tumors (Figure 3). Molecular Signature Database (Subramanian et al., 2005) was used to select the sets of target genes for Notch and Wnt pathways (see File S3). Among several available modules, we chose the ones having the best differential activity score between metastatic and non-metastatic samples for computing Notch and Wnt pathway activities. For p53 pathway activity, we used a set of known p53 primary targets (Kannan et al., 2001).

### 3.2. Dysregulated signaling pathways in bladder cancer

**Figure 4:**
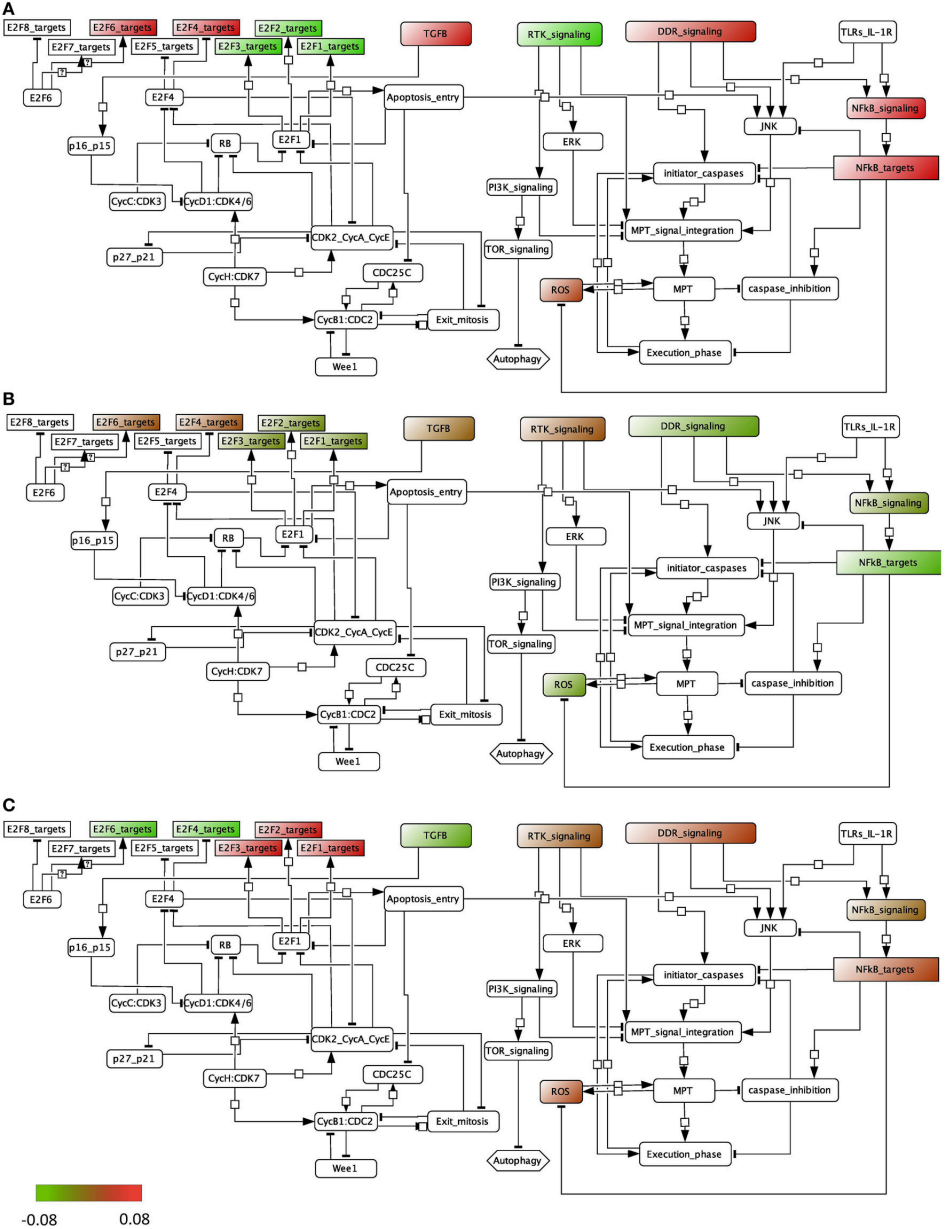
**Representation of the module activity of bladder dataset (Lindgren) onto a signaling network that is drawn from literature known facts and that illustrates the module activity for (A) normal samples, (B) superficial tumors, and (C) invasive tumors**.

We performed the ROMA analysis on a transcriptome dataset of bladder tumors with clinical information about the stage of the tumors (Lindgren et al., 2010). Two groups of samples were selected for comparison, invasive and superficial. Normal samples are also provided (details can be found in File S1). The modules of genes chosen for this analysis are those that are known to be frequently dysregulated in this cancer and that include, among others, cell cycle and apoptotic pathways (see File S2). Inside each module, the genes that are known to be representative of the activity of the module are specified as positive contributors of the module, e.g., E2F1, E2F2, and E2F3 are assigned a positive sign in the module E2F, whereas RB1 is assigned a negative weight. The modules that appear in the analysis are the ones for which at least 8 genes are found in the dataset. We plotted the module activity scores for which the L1 *p*-value was lower than 0.05 onto an influence network (Figure 4) for the three cases: normal samples, superficial tumors, and invasive tumors. The influence network was drawn using CellDesigner software with connections extracted by manual literature mining. We also plotted the module NF-KB signaling that has a *p*-value of 0.12, knowing that the activity of this module cannot be as trusted as the others.

We find that in normal samples and superficial tumor samples, the activity for the modules of the E2F1, E2F2 and E2F3 target genes is lower than in invasive tumors, as opposed to the target genes of the inhibitory transcription factors E2F4 and E2F6. This is in accordance with what is expected. Indeed, in the invasive group, tumors show a higher proliferation rate. Also, TGFb activity is lower in the invasive group than in the superficial one. Interestingly, the activity of the death signaling pathway (DDR signaling) is high in normal samples, lower in superficial tumors and start to be higher again in invasive tumors. RTK signaling activity, representing growth factors, is low in normal samples but is found high in both tumor groups. Indeed, genetic alterations in the EGFR, FGFR3, and RAS pathways are typical of tumor initiation and progression in bladder.

### 3.3. Estimating activity of EWS/FLI-1 chimeric transcription factor in ewing sarcoma

We tested ROMA algorithm on transcriptome time-course measurements performed on Ewing sarcoma inducible cell lines after EWS-FLI1 silencing and re-expression (Tirode et al., 2007; Stoll et al., 2013). EWS-FLI1 is a chimeric transcription factor specific to Ewing sarcoma disease and responsible for a tumorigenic phenotype. Different studies have reported opposing transcriptional activity of EWS-FLI1 whether it binds to transcriptional co-activators (Fuchs et al., 2003) or transcriptional co-repressors (Sankar et al., 2013). Since EWS-FLI1 functions as both an activator and an inhibitor, the simple average expression of its target genes does not reflect its active/inactive state (see boxplot in File S1). Instead, weights obtained when applying ROMA to the expression matrix of target genes provide an appropriate measure of EWS-FLI1 activity (see File S4).

**Figure 5:**
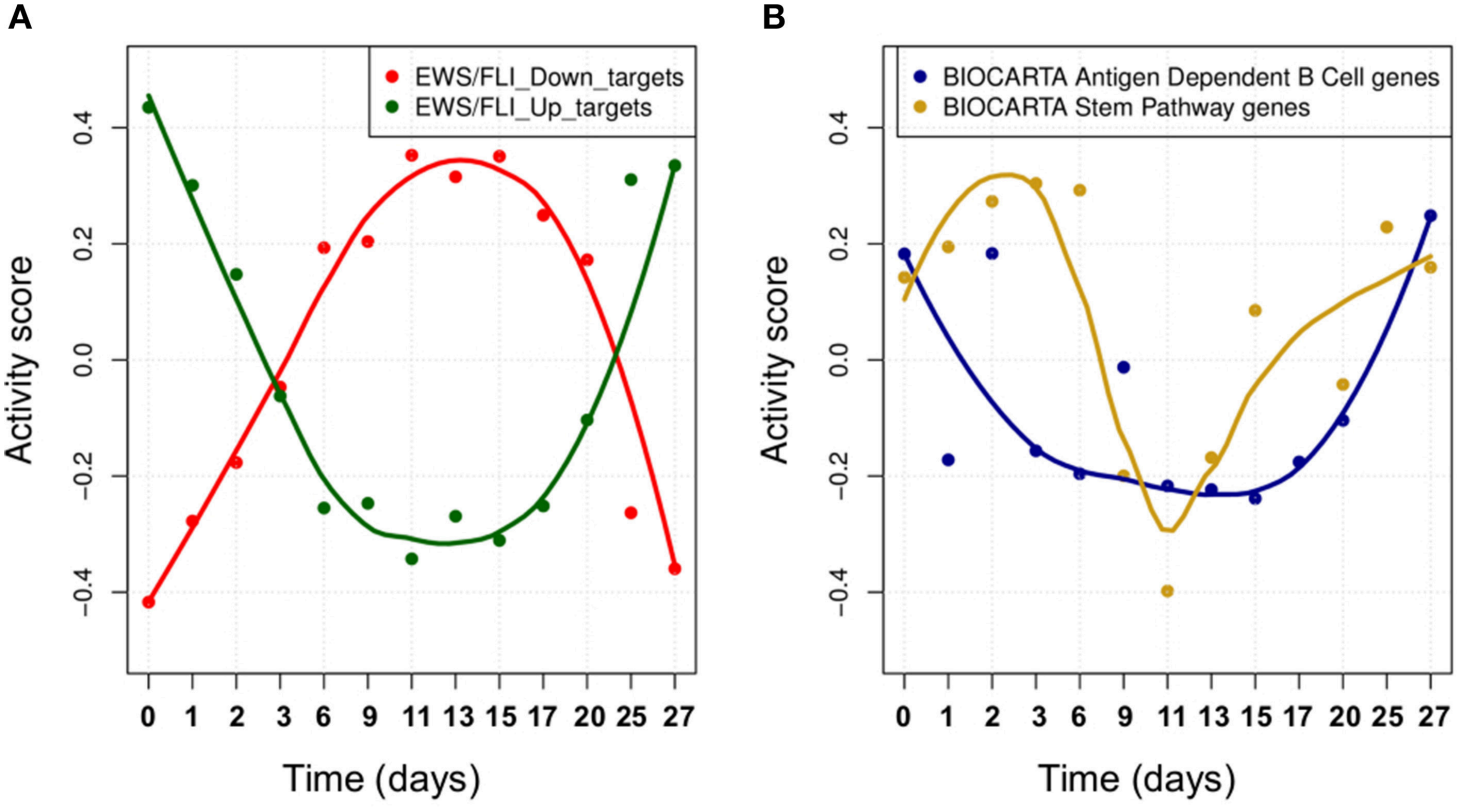
**(A)** The activity scores of both up and down-regulated target sets during EWS-FLI1 inhibition (days 0–10) and rescue (days 10–27) time series experiments. EWS-FL1 time-course related to the dataset was measured and reported in Figure 3A of (33). **(B)** Smoothed temporal activity profile for two overdispersed pathways found by ROMA in the analysis of time series expression profile after inhibition of EWS-FLI1.

We studied the effect of EWS-FLI1 on a predefined signature of dysregulated genes (Hancock and Lessnick, 2008) by computing the activity score of this set of targets over time. First, ROMA analysis was performed for the whole set of genes. In this case, the sign of the weights for some target genes was specified according to a priori biological knowledge about the regulation of up and down targets. Secondly, the same analysis was performed by splitting the initial signature in two separated modules for the predicted up and down-regulated targets. Among the three tested modules, the whole signature target set showed the most significant overdispersion pattern across time points, with *L*_1_ = 0.52 (*p*-value = 0.001). ROMA analysis using down-regulated targets gave a better overdispersion signal compared to up-regulated targets (see detailed results in File S1). We expected the activity scores of the EWS-FLI1 set of targets to show modulation of the expression of targets of EWS-FLI1 over time. Results confirmed that the activity scores of both up and down-regulated target sets properly reflected the dynamics of EWS-FLI1 expression during the inhibitory (*t* = 0−10 days) and rescue (*t* = 10−27 days) time series experiments (Figure 5A). Instead, the average expression of the same set of targets did not show modulation across the time points.

We tested whether the expression of other modules than EWS-FLI1 targets showed a significantly overdispersed pattern upon EWS-FLI1 inhibition and reactivation. This could reveal relevant biological functions affected by EWS-FLI1 expression. ROMA analysis was performed on the EWS-FLI1 transcriptome time-series using a large collection of predefined signaling pathways from Molecular Signature Database (MSigDB Liberzon et al., 2011). In this example, we used a subset of MSigDB limited to the pathway definitions imported from KEGG (Ogata et al., 1999), REACTOME (Croft et al., 2014), BIOCARTA (Nishimura, 2001) pathway databases. To these sets, we added 59 definitions of modules from Atlas of Cancer Signaling Network (ACSN) (Kuperstein et al., 2015) and the set of potential transcriptional targets of EWS/FLI-1 chimeric oncogene (Hancock and Lessnick, 2008). In total, this resulted in 1121 modules. Out of all these modules, 23 had significant overdispersion in time series measurements with *p*-value < 0.05 (see File S5). For these modules, we distinguished two different kinetics in their response to EWS-FLI1 expression reflected by their activity score, one having switch-like response similar to EWS-FLI1 signature targets and a second one similar to a pulse-like response (Figure 5B).

### 3.4. Detecting overdispersed pathways in single-cell RNAseq data

Application of module activity estimation is particularly interesting to determine molecular pathways contributing to the non-genetic heterogeneity of cell populations in the context of single cell transcriptomics data analysis (Fan et al., 2016). In order to demonstrate that ROMA can be used to detect overdispersed pathways in single cell transcriptomics data, we applied it to a set of 372 individual cell transcriptomic profiles measured in several time points after induction of differentiation in a skeletal myoblast cell culture (Trapnell et al., 2014).

The collection of gene sets used for this example was taken as in the previous section. ROMA has detected a number of overdispersed pathways (many more than in the previous examples) revealing major biological functions contributing to the cell-to-cell transcriptome variation. As expected, clustering overdispersed pathways according to their module activity score profiles (see Supplementary Materials) distinguished a large cluster of signatures related to cell cycle and closely related DNA replication and DNA repair. A large cluster of 50 signatures mixed modules related to apoptosis, respiratory electron transport, TCA cycle and various metabolism and catabolism-related modules. A cluster of 10 signatures was related to translation. Another cluster of 16 signatures contained modules related to transcription, mRNA splicing and mRNA processing. Relatively small cluster contained six signatures related to glucose transport and, surprisingly, metabolism of non-coding RNA. Two smaller clusters included five gene signatures related to extracellular matrix organization, and muscle contraction together with cardiomyopathy (which is probably more specific to the cellular function of myoblasts).

**Figure 6:**
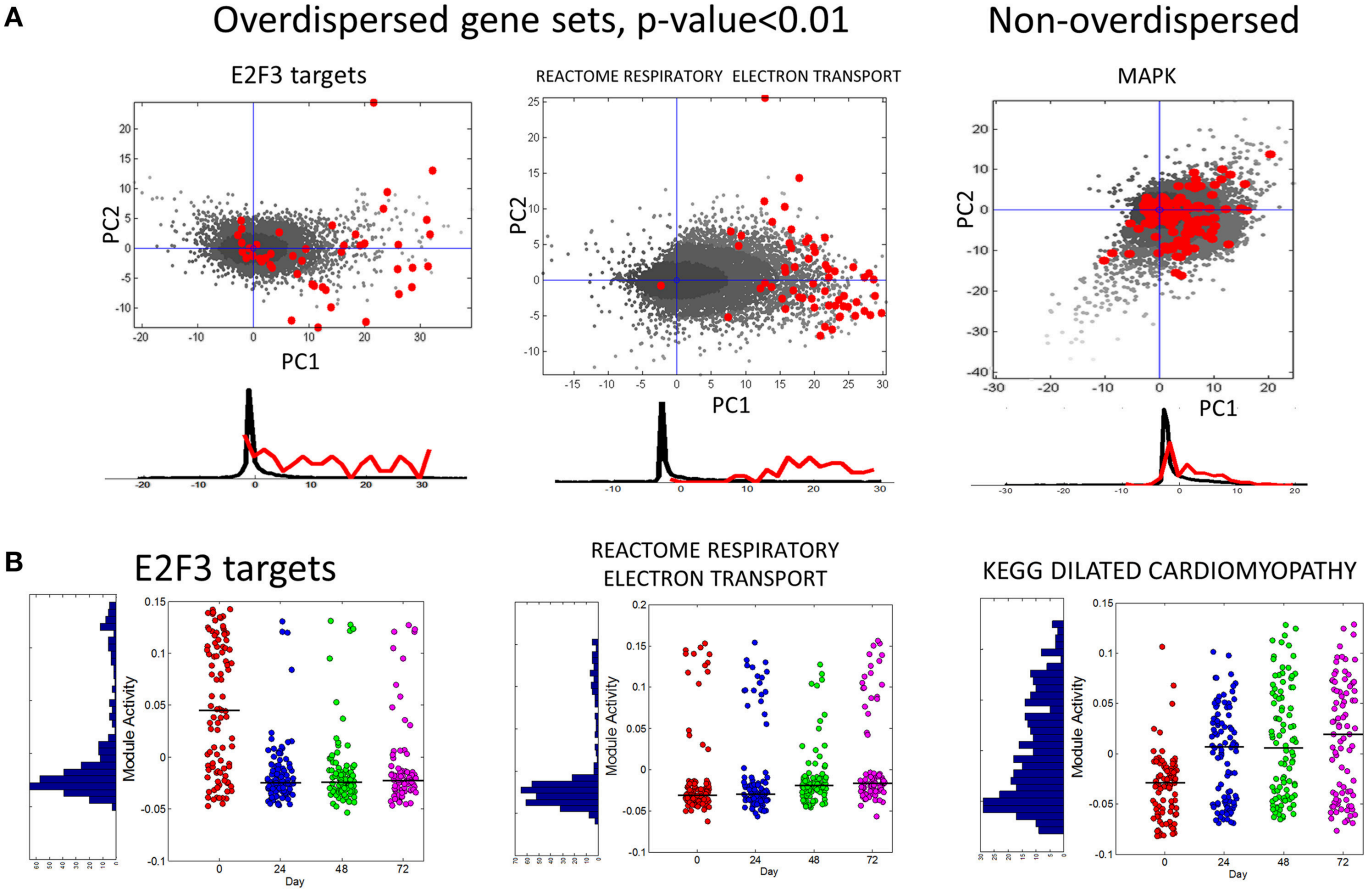
**(A)** Examples of overdispersed and non-overdispersed pathways in single-cell RNA-Seq data. Red points are the genes of the pathways, shown in the projection on the first two principal components computed for these points. Black points show the global distribution projected in the first two principal components of the pathway. Below the scatterplot, the histogram of gene projections on the PC1 is shown separately for the genes in the pathway (red) and for the global distribution (black). **(B)** Module activation score in single cells. The x-axis corresponds to four time points (T0-T72). The black line shows the median module activation score within the same time point. On the left of the graph the histogram of module activation scores for all cells in all time points is shown.

In Figure 6A we show several examples of overdispersion pattern observed in the single-cell RNASeq dataset. We observed that most overdispersed modules obtained high score due to a systematic shift with respect to the global gene distribution, such as the leftmost E2F3_TARGETS signature in Figure 6A. In Figure 6B we show the profiles of module activity scores across all cells, ordered in time. E2F3_TARGETS signature from ACSN pathway database probably marks the cells in the active proliferation state. One can see that the number of proliferating cells drops at the time point T24 when compared to the time point T0. However, there remains a significant number of proliferating cells after T24. Interestingly, the modules can be classified into those showing clear bimodal distribution of activity scores and those having unimodal distribution (e.g., see the KEGG dilated cardiomyophathy profile in Figure 6B). One can observe also that the variance of module activity scores might vary significantly from one time point to another (see the same profile on Figure 6B).

Note that in all of the four analyses presented above, we have found a large set REACTOME_OLFACTORY_SIGNALING_PATHWAY overdispersed. Olfactory receptors are known to be a common confounding signal in many mutation profiling analyses (Lawrence et al., 2013). It seems that this is also reflected in pathway overdispersion analysis, based on transcriptomic data of normal or cancer cells. We are not aware that this phenomenon was described before.

## 4. Discussion

Quantifying the activity of biologically related modules is a widely exploited strategy to extract biological information from high-throughput data. In the analysis of genomic data, using gene sets as aggregated variables can help to capture biological information that is otherwise undetectable by focusing only on individual genes. We introduced the ROMA algorithm which deals with this problem of quantifying the activity of modules by fast and robust computation of the simplest linear model of gene regulation based on computing the PC1 of the expression data matrix and estimating the statistical significance of such approximation.

We tested ROMA on a first case study to quantify the activity of Notch, Wnt and p53 pathways in metastatic and non-metastatic tumors from human colon cancer transcriptome data. Unlike single gene expression analysis, the ROMA algorithm has effectively shown the involvement of these pathways in the metastastic process by detecting their differential activity. In this study, the sets of downstream transcriptional targets reflect the activity of the associated pathways better than any individual gene involved in the signaling cascades. In similar gene set analysis, ROMA can be considered as a powerful algorithm to detect coordinated but small changes of several genes in a pathway.

In our second example ROMA was used to map the expression profiles of bladder patients on an influence graph that recapitulates the molecular interactions between different pathways. The information extracted from the data correlates to what is known about the tumor progression in bladder cancer. To complete the analysis, it would be possible to translate the influence network into a logical model. This would consist in associating to each module (equivalent to a variable of the model) a logical rule linking all of his inputs with the logical operators AND, OR, and NOT. For instance, ROS would be written as follows: ROS = MPT AND NOT NFkB_targets. Thus, if the influence network was to be translated into a logical model and simulated for each patient profile (set of mutations or genetic alterations known for the genes included in the model) with accompanying clinical information (stage of the tumor), we would expect to see the solutions of the simulation, referred to as stable states, of an invasive patient with active E2F1, E2F2, and EF3 target variables (equal to 1) whereas the stable states for patients with superficial tumors with these variables equal to 0. The data analysis performed with ROMA is also one way to assess that the logical rules are in accordance with the dataset that is studied and thus that the model represents correctly the dynamics of bladder tumorigenesis. Another possible use of ROMA in the context of network modeling can be in the selection of the pathways of interest to include in the model. Constructing a structural model of a specific complex molecular process can be based on literature information combined with an exploratory analysis of pathway databases to identify those pathways that are active or inactive in a particular cellular condition.

In the third example, we described the application of ROMA in quantifying transcriptional activity of targets of EWS-FLI1 from time-course measurements. Since this oncogenic TF can have both inhibitory and activating properties, ROMA analysis was performed first for the whole set of known target genes and secondly by splitting the set in two separated modules for the up and down regulated targets. The whole signature target set was the most significantly overdispersed. This is consistent with the fact that a larger set of co-regulated genes, regardless of the regulation sign, is expected to generate a stronger overdispersion signal. This is an advantageous property of ROMA compared to other gene set testing methods, such as GSEA, that estimate the significance of enrichment score by considering separately the positively and negatively scoring gene sets. Also, several TFs can have both inhibitory and activating function; ROMA can be applied without information about the sign of the TF effect on its targets. In time series data, the scores calculated on the sets of targets can give information on the kinetics of the transcriptional response. The activity scores of targets reflect the dynamics of EWS-FLI1 expression during the inhibitory and rescue experiments.

In the fourth example, ROMA is applied to detect overdispersed pathways in single cell transcriptomics data. This is particulary interesting application of unsupervised ROMA approach, because it potentially allows quantifying the non-genetic heterogeneity of a cell population on pathway level. Multiple gene sets have been shown to be overdispersed in this case: therefore, clustering them based on the activity profiles over the cell population helps identifying the major functional aspects contributing to cell-to-cell variance.

In many studies ROMA can be applied to unravel the effective status of a TF protein from the expression of its target genes. The predicted activity values can be validated experimentally. if the active form of a transcription factor or other factor is known and can be measured (i.e., by mass spectrometry measurements), or the factor represents a measurable phenotypic read-out (such as cell growth or age).

Oncogenes and tumor suppressor regulatory genes, such as p53, often carry mutations in their DNA sequences. However, such DNA changes do not always have a clear effect at the phenotypic level. On the other hand, the function of oncogenes or tumor suppressors can be compromised by other mechanisms than DNA mutations, like for example alterations in DNA methylation. Computing activity score of transcriptional target sets is a useful method to assess the active or inactive status of regulatory oncogenes or tumor suppressors. We can also imagine to label tumor samples in a more reliable manner by relying both on the targets activity score and on DNA mutations. Our previous study shows that the estimated activity of p53 in tumor samples is better associated to the clinical outcome than expression or mutation status of p53 alone (unpublished data). Recent advances in chromatin immunoprecipitation with next-generation DNA sequencing (ChIP-Seq) have provided large collections of detected TFBSs with high sensitivity that facilitate the comprehensive annotation of TF targets sets.

The idea of applying ROMA in order to investigate the effect of regulatory molecules can be generalized in order to study other classes of regulators, such as kinases, phosphatases, microRNAs, etc. The availability of large-scale proteomics and phosphoproteomics data gives unprecedented knowledge about post-transcriptional and post-translational regulation happening in the cell. The ROMA algorithm can be applied to analyze quantitative phosphoproteomics profiles and identify overdispersed patterns of predefined sets of proteins sharing common phosphorylation sites. By exploiting this information it would be possible to infer active or inactive kinases/phosphatases.

Multiple types of analyses using ROMA can be performed in order to explore microRNA regulation. First, microRNA genes appear often organized in genomic clusters that are not randomly composed, meaning that this clustered structure is evolutionary conserved and is likely to be related to miRNAs coordinated regulatory action. Comparing expression level of clustered miRNAs in different conditions, the variation in the abundance of each individual miRNA of the cluster can be weak and not detectable by standard statistical hypotheses testing applied to individual miRNA expression levels, while the overdispersed expression pattern of the entire cluster can produce a statistically significant signal and reveal its differential activity.

ROMA can also be useful for the identification of microRNA regulation by expression analysis of target genes. The module approach is particularly suitable to infer miRNA regulatory effect from target expression profiles, since miRNA effect is subtle at the level of individual target but affects a large number of genes (Martignetti et al., 2015).

ROMA can be used in combination with unsupervised methods for metagene extraction from omics data such as Independent Component Analysis (ICA) for helping component interpretation (Zinovyev et al., 2013; Biton et al., 2014).

In the future it would be interesting to generalize the linear model of ROMA method onto a non-linear case, through application of non-linear versions of principal component analysis such as principal curves (Gorban and Zinovyev, 2001; Gorban et al., 2008) or principal trees (Gorban and Zinovyev, 2009). Indeed, distributions of gene expression profiles are demonstrated to contain non-linearities (Drier et al., 2013) and branching points. For example, a variant of principal curve approach was suggested in Trapnell et al. (2014) in order to recapitulate the non-linear dynamics of myoblast differentiation. Non-linearity leads to the situation when there exists no one single set of genes contributing the most to the definition of module activity: this set will depend on a particular region of the gene expression space. This will complicate the interpretation of the module activity: however, many ideas introduced in ROMA (estimating statistical significance of overdispersion, robust modification of non-linear PCA, etc.) will remain applicable.

To conclude, we prove that ROMA is useful when applied to different biological case studies. ROMA will contribute to the set of tools routinely applied in systems biology according to the application examples outlined before. In the future, we will provide a Graphical User Interface to facilitate the use of the ROMA algorithm, in the form of a Cytoscape app (Smoot et al., 2011; Saito et al., 2012).

## Author contributions

LM, LC, EBa, and AZ designed and implemented the methodology. EB packaged the code and worked on improving the methodology. LM, LC, and AZ has provided the examples of methodology use. All authors have read and worked on the manuscript.

### Conflict of interest statement

The authors declare that the research was conducted in the absence of any commercial or financial relationships that could be construed as a potential conflict of interest.

## Supplemental data

File S1 **Supplemental Material about the described examples and results**.

File S2 **Modules gmt file used in bladder cancer data analysis**.

File S3 **Sets of target genes for p53, Notch and Wnt pathways from Molecular Signature Database**.

File S4 **Weights for up-regulated genes, down-regulated genes and full signature obtained by ROMA to the expression matrix of target genes**.

File S5 **Modules gmt file used for the two last examples. The pathway definitions come from KEGG, Reactome, Biocarta and ACSN**.
