# Supplementary material for: ROMA: Representation and Quantification of Module Activity from Target Expression Data
Source: Front Genet. 2016 Feb 19;7:18. doi: 10.3389/fgene.2016.00018 (PMC4760130; doi:10.3389/fgene.2016.00018)
Supplement: File S1 — Supplemental Material about the described examples and results. [file FileS1.docx]

**Supplementary information**

**Examples**

1. Notch, Wnt and p53 pathways in colon cancer 1

2. Dysregulated pathways in bladder cancer 2

3. Ewing sarcoma results 5

4. Single-cell transcriptome analysis by ROMA 7

## 1. Notch, Wnt and p53 pathways in colon cancer

*Description*:

121 patients are analyzed with both transcriptomics data and clinical information about the metastatic status.

The patients are separated into two groups: 19 metastatic (M1) tumors and 102 non metastatic (M0) tumors.

Data from: Muzny, D. M. *et al.* Comprehensive molecular characterization of human colon and rectal cancer. Nature 487, 330–337 (2012).

Differential expression analysis of single genes involved in Wnt and Notch and p53 signaling pathways did not show significant changes between metastatic and non-metastatic tumors.


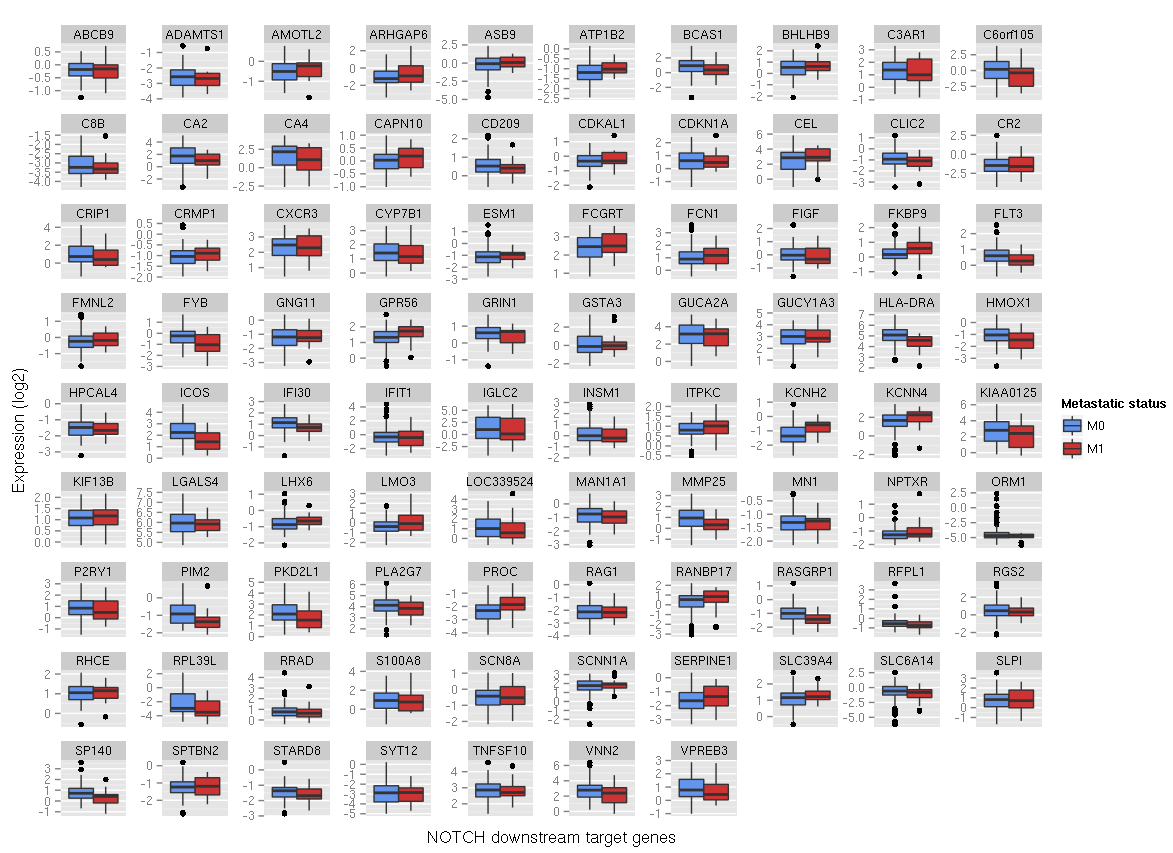


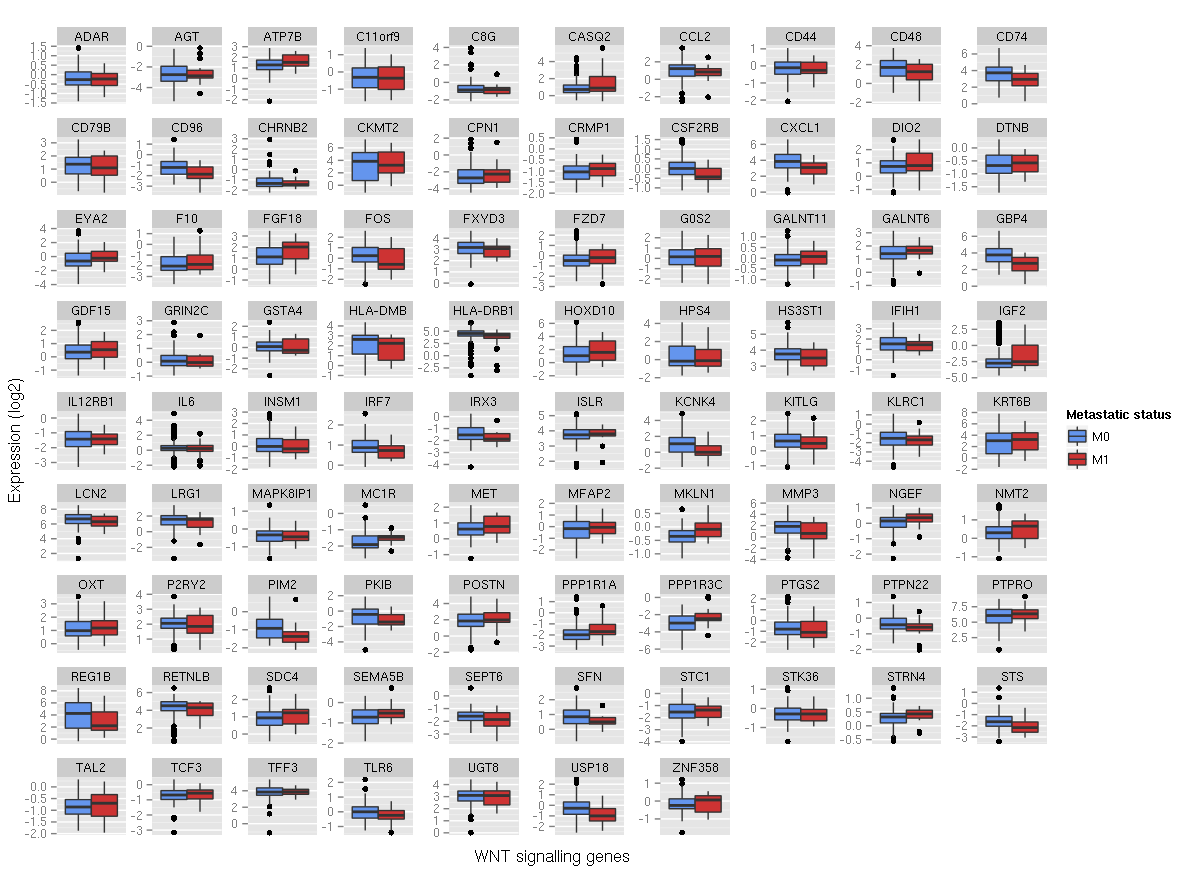


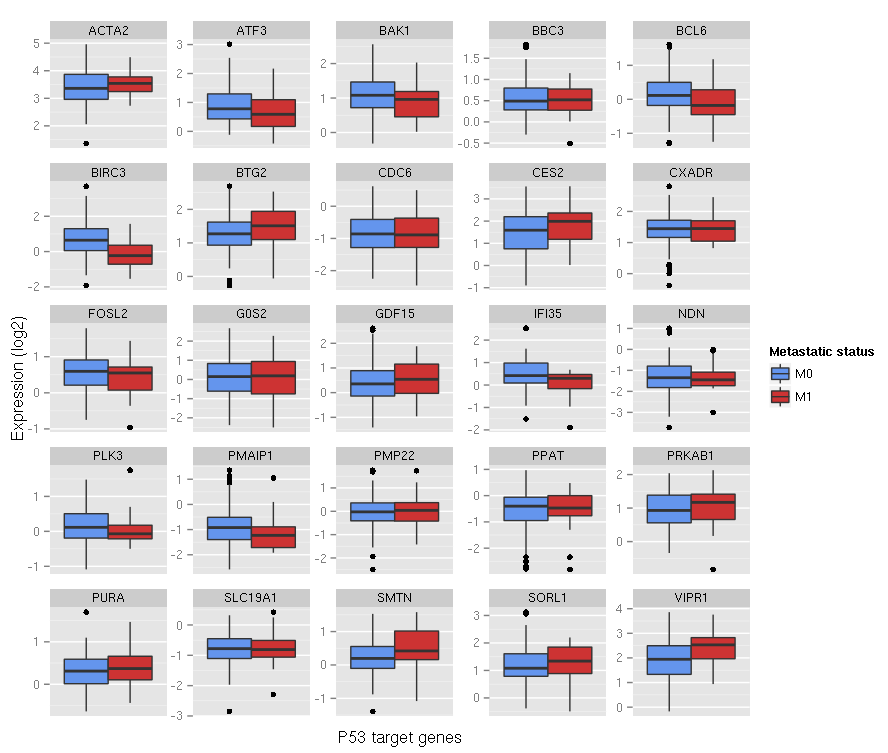


## 2. Dysregulated pathways in bladder cancer

***Data description***

101 patients are analysed with both transcriptomics data and clinical information about the stage of the tumours.

The patients are separated into two groups: 67 superficial (Ta, T1) tumours and 34 invasive (>=T2) tumours.

12 normal samples are also provided.

**Citation:**

Lindgren D, Frigyesi A, Gudjonsson S, Sjödahl G et al. Combined gene expression and genomic profiling define two intrinsic molecular subtypes of urothelial carcinoma and gene signatures for molecular grading and outcome. Cancer Res 2010 May 1;70(9):3463-72. PMID: [20406976](http://www.ncbi.nlm.nih.gov/pubmed/20406976)

[Query DataSets for GSE19915](http://www.ncbi.nlm.nih.gov/sites/entrez?db=gds&term=GSE19915%5BAccession%5D&cmd=search)

***Construction of the influence network***

We constructed the network in CellDesigner*. The network is an influence network where nodes are modules that represent genes/pathways that are often altered in cancer, and edges are positive or negative influences that these genes/pathways have on each other. The network is hand-made and is based on known facts extracted from literature.

* <http://www.celldesigner.org/>

***Module description***

We defined lists of genes that represent each node of the network in the gmt file (File S2). We consider each node as a module.

The genes composing a module are chosen such that they illustrate the meaning of the module. For transcription factors, we choose the target genes of the transcription factor. For a pathway, we choose genes that participate in the pathway. We based these lists for existing databases such as ACSN (1), KEGG (2), and REACTOME (3).

Since in the pathway, there might be both inhibitors and activators in the list, we assign a positive weight (+1) to the activators and a negative weight (-1) to the inhibitors.

(1) <http://acsn.curie.fr/>

(2) <http://www.genome.jp/kegg/pathway.html>

(3) <http://www.reactome.org/>

***Computation of the module activity score***

We plotted the expression for each module that is computed with ROMA using both BiNoM plugin * of Cytoscape ** (for importing the CellDesigner network into Cytoscape) and the vizmapper of Cytoscape for assigning colours to the score calculated by ROMA for each module.

The modules that are identified are the modules for which at least 8 genes were found in the dataset. Among the 39 modules described, the activity of 13 is computed. The modules RB and MPT_signal_integration have a high p-value, thus are not plotted on the influence graph of Figure 2 in the main text.

The activity of each module is provided in the table below for normal samples, superficial tumors and invasive tumors, along with the standard deviation.

The parameters that we used to perform the analysis are the following:

outputFolder= RBE2F_Lindgren/

dataFile= lindgren_data_mod.txt

sampleFile= lindgren_clin.txt

moduleFile= modules_signed.gmt

centerData= false

doubleCenterData= false

fillMissingValues= -1

activitySignsFile= null

mostContributingGenesZthreshold= 1.0

diffSpotGenesZthreshold= 1.0

typeOfPCAUsage= 1

robustPCACalculation= true

robustPCACalculationForSampling= false

outlierThreshold= 2.0

typeOfModuleFile= 1

fieldForAveraging= invasiveness

fieldValueForAveraging= null

fieldForDiffAnalysis= invasiveness

fieldValuesForDiffAnalysis= superficial#invasive

minimalNumberOfGenesInModule= 10

minimalNumberOfGenesInModuleFound = 8

* <http://binom.curie.fr/>

** [www.cytoscape.org/](http://www.cytoscape.org/)

## 3. Ewing sarcoma results

Boxplot showing that the simple average expression of EWS-FLI1 target genes does not reflect its active/inactive state.


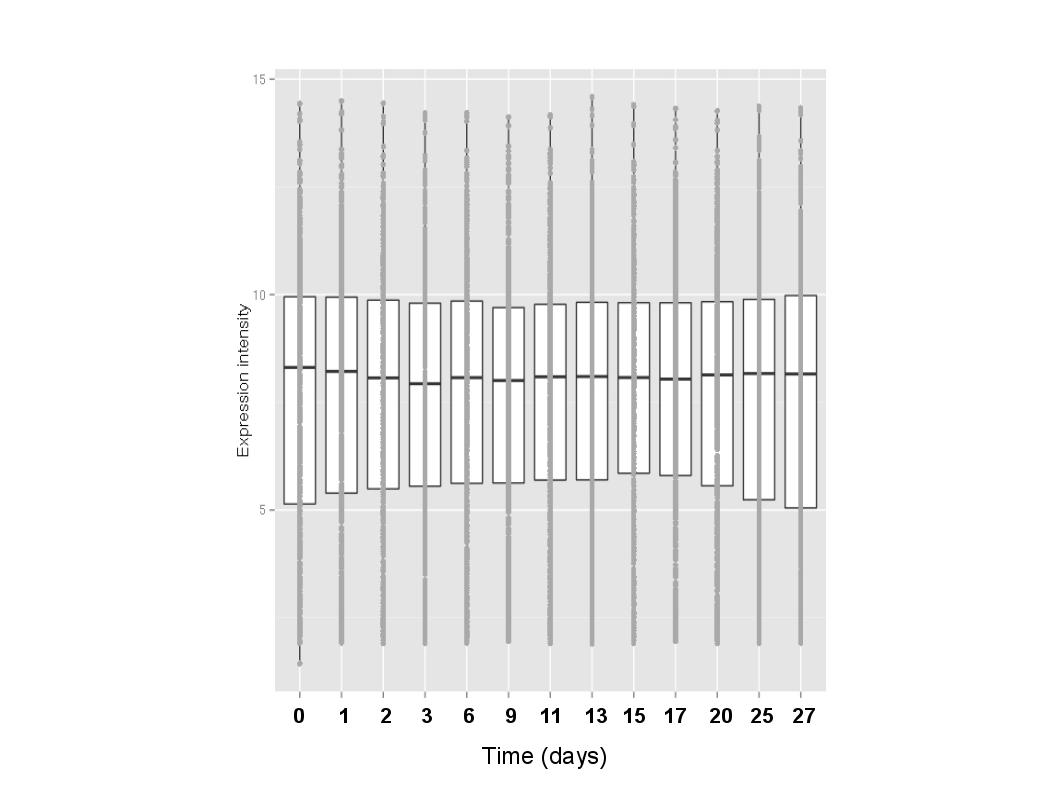


| MODULE | L1 | L1 _pv | L1/L2 | L1/L2_pv | NUMBER_OF_GENES |
| --- | --- | --- | --- | --- | --- |
| Lessnick_EWS-FLI_Down_signature | 0.58 | 0.005 | 3.02 | 0.142 | 280 |
| Lessnick_EWS-FLI_Up_signature | 0.47 | 0.087 | 2.89 | 0.12 | 492 |
| Lessnick_EWS-FLI_All_signature | 0.52 | 0.001 | 2.96 | 0.06 | 769 |

## 4. Single-cell transcriptome analysis by ROMA

Correlation graph depicting connections, at the level of correlations between module activities profile, for 213 overdispersed gene sets found in single-cell transcriptomics data on induction of myoblasts differentiation. Major cluster of gene sets are annotated.
